# Contribution of social support and partner communication quality to mental health among combatants’ partners

**DOI:** 10.1080/08995605.2025.2470480

**Published:** 2025-03-19

**Authors:** Liat Kulik, Anita Zorchinsky

**Affiliations:** a School of Social Work, Bar Ilan University, Ramat Gan, Israel; b School of Behavioral Sciences, Netanya Academic College, Netanya, Israel

**Keywords:** Electronic communication, face-to-face communication, combatants’ partners, Swords of Iron War, mental health

## Abstract

This study investigated the relationship between social support and communication quality among combatants and their partners, and the partners’ mental health during Israel’s Swords of Iron War against Hamas. Mental health was assessed through emotional, social, and psychological dimensions. Communication quality was evaluated by assessing both positive and negative aspects of electronic and face-to-face channels. The sample included 201 women in various relationship statuses with men who were recruited as combatants: married, cohabiting, and in a stable relationship. Most women reported positive electronic and face-to-face communication with their partners during the war. Nonetheless, nearly 20% mentioned an increase in the negative aspects of face-to-face communication compared to pre-war conditions. Negative communication correlated with poorer psychological health. No direct link was found between positive communication and mental health. However, an indirect relationship was observed, with perceived social support serving as a mediator. The psychological dimension ranked highest among the mental health dimensions, followed by the social dimension, with the lowest ranking found for the emotional dimension.

**What is the public significance of this article?—**A recent Israeli study during the Swords of Iron War reveals that although communication between military couples is mostly positive, 20% experienced increased negative patterns, including criticism and intolerance. This highlights the need for short-term intervention programs to improve communication skills among military couples during prolonged conflicts, aiming to enhance their mental health and prepare them for post-war life together.

## Introduction

This study explored the mental health of combatants’ female partners (hereinafter “partners”) during the Swords of Iron War between Israel and Hamas, which began in October 2023. War-related stressors can not only negatively impact the mental health of combatants, but also that of their partners, via interpersonal communication and shared experiences (Cigrang et al., 2014; Karney & Crown, 2010). The research examined how perceived social support (hereinafter “social support”) and communication quality during the war may be associated with partners’ emotional, social, and psychological mental health. Social support is known to benefit mental health during stressful situations (Skomorovsky, 2014). The quality of communication between combatants and their partners may facilitate adaptation to war-related psychological distress (Sayers & Rhoades, 2018; Seidel et al., 2014). However, in some cases, war-related stress can contribute to negative communication patterns that can harm the partner’s psychological well-being (Knobloch et al., 2013; Zamir et al., 2020).

Given the significant role of interpersonal communication in mental health, this study aimed to fill the gap in research on this issue, particularly within the context of war. The findings may afford practical recommendations for effectively managing communication during wartime, benefiting both combatants and their partners. The importance of social support and positive communication in promoting mental health is universally recognized, especially during times of war. Human nature dictates that individuals seek support during stressful times, and this need may be fulfilled through effective communication between partners. The findings may thus be applicable to other war situations, highlighting the universal need for connection and understanding amidst conflict.

### Theoretical framework: Conservation of resources (COR) theory

The Conservation of Resources (COR) theory (Hobfoll, 1989) forms the basis for the current research. COR posits that individuals strive to maintain their resources, and the loss of these resources leads to stress, impacting mental health (Hobfoll et al., 2018). Resources are broadly defined as personal or environmental characteristics that contribute to individual well-being, such as personal traits, time, assets, energy, and social interaction. This approach has been applied to various threatening situations, including wartime scenarios (Hobfoll et al., 2012; Vinokur et al., 2011). The theory emphasizes the dynamic nature of resource conservation, noting that efforts to preserve or replace lost resources can cause additional stress. COR theory also highlights how the context of resources changes during stress development, such that their value and impact may shift. For instance, while positive communication between combatants and their partners can be a valuable resource both during routine times and in times of war, its nature during war may shift to include negative aspects such as criticism, blame, and disagreements. As a result, both parties involved in the interaction may be adversely affected. This negative communication can arise from the stress and trauma associated with combat, but also from the experiences of partners who must cope alone with the stress of war and sometimes also with challenges of raising their children (Larsen et al., 2015).

Based on COR theory, we propose a research model that examines the relationship between social interaction resources – specifically social support and the quality of communication among partners of combatants during the Swords of Iron War – and the partners’ mental health indicators.

### The hypothetical research model: Social interaction resources and mental health

**Figure 1.**
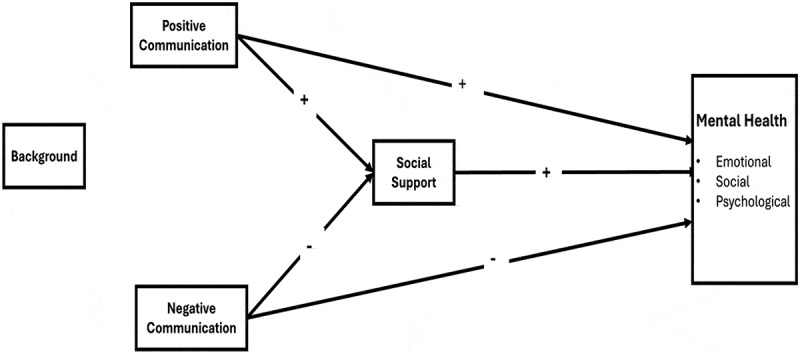
Hypothetical model.

In the hypothetical research model (Figure 1), the mental health of combatants’ partners serves as the dependent variable, reflecting a contemporary, multidimensional construct. Social interaction resources (social support and spousal communication quality) serve as independent variables.

#### The outcome variable: Mental health

Historically, mental health was defined as the absence of psychopathology (Jahoda, 1959; Keyes, 2005). Mental health is considered a central aspect of well-being. It encompasses psychological, social, and emotional dimensions that reflect its broader definition (Keyes, 2002). This broader concept encompasses an individual’s ability to cope with stress, derive satisfaction from social interactions and work, and contribute to society (World Health Organization, 2004).

This comprehensive view of well-being stems from two philosophical traditions: hedonic and eudaimonic (Deci & Ryan, 2008; Ryff, 1989). The hedonic approach, rooted in the philosophy of Democritus, emphasizes life satisfaction and positive emotions (Keyes, 1998). The eudaimonic approach, based on Aristotelian thought, focuses on life meaning, self-actualization, and optimal functioning in personal and social spheres (McMahan & Estes, 2011; Waterman, 1993). The eudaimonic tradition highlights social well-being including social integration and contribution, and psychological well-being encompassing self-acceptance, personal growth, and autonomy (Keyes, 2002, 1998). By integrating hedonic and eudaimonic perspectives, this research adopts a holistic view of mental health, considering emotional, social, and psychological dimensions of the construct (Keyes, 2002).

#### The independent variables: Social interaction resources

The main independent variables reflect the social interaction resources of combatants’ partners, manifested primarily through social support and communication quality. These resources, along with relevant background variables, are hypothesized to be related to the partners’ different mental health dimensions. In the proposed research model, we hypothesize both direct and indirect effects between the independent variables and the various dimensions of mental health. The model aims to clarify how the social interaction resources of combatants’ partners are related to their mental health across different dimensions.

### Direct effects

*Social support and mental health*. Social support is a crucial environmental resource in various stress-coping models, with the potential to mitigate negative outcomes (for a meta-analysis, see Harandi et al., 2017). Its contribution to individual well-being is particularly significant when perceived as aiding in daily coping, providing structure, and promoting adaptation during wartime (Simsir & Dilmac, 2021; Wang et al., 2018). During war, social support can take diverse forms: emotional support which offers encouragement, comfort, and shared experiences to prevent loneliness and helplessness; and instrumental support which provides practical assistance for task completion and emergency organization (Mankowski et al., 2015). Social support can include informational support, which provides necessary information and guidance to help individuals make decisions and navigate complex situations during war (Hobfoll et al., 2007). Community support also plays a vital role, fostering peer networks and enhancing trauma competence among community members to maintain social cohesion and resilience (Norris et al., 2008).

Based on extensive research demonstrating the link between social support and mental health (for meta-analyses, see Almedom, 2004; Zalta et al., 2021), we hypothesized:

H1: A positive correlation will be found between social support and mental health, with higher levels of social support related to better mental health.

*Communication between partners during wartime*. High-quality communication between combatants and their partners during wartime serves as a vital resource for social interaction, providing significant emotional and psychological benefits to both parties (Knobloch et al., 2018). The limited research on this topic draws primarily from studies of partners of American combatants in Iraq and Afghanistan (Balderrama-Durbin et al., 2018). Two main criteria for classifying partner communication during wartime are the channel of communication (electronic or face-to-face) and the quality of communication (positive or negative) (Carter & Renshaw, 2016; Rossetto, 2013).

Modern technology facilitates electronic communication channels, including mobile phones, video calls, and social media, which are crucial for combatants during deployment, allowing them to stay connected with their loved ones. Research has highlighted the importance of these communication channels for maintaining relationships and emotional well-being during deployment (Carter & Renshaw, 2016). When combatants are deployed during wartime, face-to-face communication, which comprises a fundamental form of interpersonal interaction, occurs only during the combatants’ leave periods. This type of communication can be manifested verbally, nonverbally, and through expressions that convey intimacy. Studies indicate that face-to-face interactions during leave periods are essential for fostering and maintaining couplehood (Knobloch et al., 2018).

Two dimensions related to the nature and quality of communication can be identified within each of these channels: positive communication which involves warm words, sharing feelings, and encouragement, and negative communication which includes criticism, blaming, and disagreement. These dimensions are not opposite ends of a single spectrum. Rather, they are distinct aspects that can coexist in communication patterns (Sanford, 2010).

Research has revealed that positive communication may enhance psychological resilience, coping ability, and adaptation to the situation by providing support through mutual encouragement, problem-solving, and expressions of love and understanding (Houston et al., 2013; Knobloch et al., 2016). Based on these findings, we hypothesized:

H2: *A positive correlation will be found between positive communication (electronic and face-to-face) and mental health, with higher levels of positive communication related to better mental health among combatants’ partners*.

Conversely, negative communication may serve as an additional stressor for combatants’ partners, potentially harming their mental health (Segrin, 2014). The negative affect accompanying such communication, combined with the strain of navigating stressors, can deplete partners’ emotional resources, which are already diminished due to the war. According to the COR theory, this depletion exacerbates stress levels and may contribute to psychological distress. Thus, we hypothesized:

H3: A negative correlation will be found between negative communication (electronic and face-to-face) and mental health, with higher levels of negative communication related to poorer mental health among combatants’ partners.

### Indirect effects

*Communication between partners, social support, and mental health*. In addition to the direct relationship between social interaction resources and mental health, we hypothesized an indirect relationship involving communication between partners across two channels and mental health, with social support acting as a mediator. This hypothesis is based on research suggesting that positive communication between partners during war may enhance their sense of social support, helping them cope with concerns for the combatant’s well-being (Skomorovsky, 2014). Given the established relationship between social support and mental health (Almedom, 2004; Zalta et al., 2021), we hypothesized.

H4: *An indirect relationship will be found between positive communication and mental health, mediated by social support. Higher levels of positive communication will be related to higher perceived social support, which in turn will be related to better mental health among combatants’ partners*

Based on research indicating a negative relationship between negative couple communication and social support (Sullivan et al., 2010), and the positive link between social support and mental health reported above, we hypothesized:

H5: *An indirect relationship will be found between negative communication and mental health, mediated by social support. Higher levels of negative communication will be related to lower perceived social support, which in turn will be related to poorer mental health among combatants’ partners.*

## Methods

### Sample and procedure

The sample included 201 female partners of combatants who voluntarily responded to a questionnaire distributed within a support group established for combatants’ partners during the Swords of Iron War. The support group included several thousands women who maintained virtual connections and occasionally met for various activities aimed at providing support and enrichment. Data were collected electronically from February to March 2024. The time required to answer the questionnaire was about 20 minutes. Completion of the questionnaire was voluntary. Approximately 80% of those approached completed the questionnaire. Ethical approval was obtained from the Ethics Committee of the Netanya Academic College, the institution where the research was conducted.

#### Participants’ background variables

Marital status: 43.8% (88) of the participants were married, 27.9% (56) were cohabiting with partners, and 28.3% (57) were in a stable relationship without cohabitation. The mean age was 28.22 (SD = 6.2). Parental status: 72.2% (145) were childless and 27.8% (56) were mothers. Religious self-identification: 51.2% (103) defined themselves as secular, 25.8% (52) as traditional, and 22.8% (46) as religious. Employment status: during the war, 77.2% (156) were employed and 22.8% (45) were not employed. Education: 35.8% (72) have a full high school education, 13.4% (27) have non-academic post-secondary education, 39.8% (80) have a bachelor’s degree, and 10.9% (22) have a master’s degree. Military service during the war: 82.5% (166) of the participants did not serve in the reserves or regular army, and 17.4% (35) served in the reserves or regular army. Place of residence: 19.4% (39) live in the north, 9.0% (19) live in the south, 50.2% (101) live in the center, and 21.4% (42) live throughout the country.

#### Combatants’ background variables

The combatants’ background variables were assessed based on their partners’ responses to the questionnaire. Military service during the war: 78.1% (157) were drafted from the beginning of the war, 16.4% (33) were drafted for a period of three months, and 5.5% (11) were drafted for less than three months. Frequency of home leave: 51.7% (104) of the participants reported that their partners come home on leave every week, 31.8% (64) reported a frequency of every two weeks, and 16.5% (33) reported a frequency of every three weeks or longer. Israel is a relatively small country, allowing some combatants in war zones to return home frequently, often for short refreshment leaves. In contrast to other countries, where combatants may face long deployments away from home, many Israeli combatants in this research sample could visit their families once a week. However, as the distribution of lives reveals, this frequency may not apply to all combatants, especially those in critical roles at the war zone.

### Research instruments

The research instruments included several questionnaires:

***The Mental Health Continuum-Short Form (MHC-SF) Questionnaire,*** developed by Lamers et al. (2011), consists of 14 items designed to assess various aspects of mental health. Participants are instructed to reflect on their experiences over the past month and rate each item on a 6-point scale, ranging from 1 (never) to 6 (every day). The questionnaire encompasses three distinct content domains: **the emotional mental health factor**, which reflects positive emotions and includes items such as “During the past month, how often did you feel satisfied with life?” (3 items); **the social mental health factor**, which reflects positive social functioning and includes statements like “During the past month, how often did you feel that you belonged to a community (like a social group or your neighborhood)?” (5 items); and **the psychological mental health factor**, which reflects psychological resilience with items such as “During the past month, how often did you feel that you had experiences that challenged you to grow and become a better person?” (6 items). Scores for each factor are calculated by averaging the responses within that factor. Cronbach’s alpha internal consistency was satisfactory across all factors: α = 0.80 for the emotional factor, α = 0.83 for the social factor, and α = 0.84 for the psychological factor.

***The Multidimensional Scale of Perceived Social Support (MSPSS)*** developed by Zimet et al. (1988) is a 12-item self-report questionnaire assessing an individual’s perception of available social support. The scale measures perceived social support from family, friends, and significant others. Participants rate their responses on a 7-point scale, ranging from 1 (strongly disagree) to 7 (strongly agree). An example item is “There is a special person who is around when I am in need.” The MSPSS yields a total score based on the three sources of support, with higher scores indicating higher levels of perceived social support. Cronbach’s alpha internal consistency of the scale was high (α = 0.94).

***The Electronic Communication Inventory (ECI)*** used in the present study is based on the Deployment Communication Inventory (DCI), developed by Balderrama-Durbin et al. (2018). The DCI is a research tool consisting of 12 items designed to assess the quality of communication between military partners during deployment periods. This adapted ECI specifically aims to assess electronic communication, while maintaining the structure of the original DCI. The inventory consists of two subscales: Positive Electronic Communication (6 items) and Negative Electronic Communication (6 items). Participants are instructed to reflect on their experiences during the deployment period and rate each item on a 5-point Likert scale, ranging from 1 (not at all) to 5 (very much so). The ***Positive Electronic Communication*** scale assesses communication characterized by comfort and supportiveness, with a sample item being “We talked about how much we look forward to seeing each other again. The ***Negative Electronic Communication*** scale assesses communication characterized by arguing and criticism, exemplified by the item “My partner criticized me for not being supportive/helpful enough during the deployment.” Scores for each subscale are calculated by averaging the responses to the respective items, with higher scores indicating greater levels of that type of communication. Cronbach’s alpha internal consistency yielded values of α = 0.86 for the Positive subscale and α = 0.83 for the Negative subscale.

***Face-to-Face Communication Inventory.*** To assess face-to-face communication between combatants and their partners, we used the Conflict Communication Inventory (CCI) developed by Sanford (2010). The original questionnaire included items measuring communication from the male partner to the female partner and vice versa. In our research we used only the 14 items that describe communication from the combatant to his partner. This subset consisted of 7 items assessing positive communication and 7 items assessing negative communication. ***Positive Communication Patterns*** reflect supportive communication styles from the combatant to his partner, as illustrated by items such as “My partner made me feel like my opinion was important. ***Negative Communication Patterns*** capture critical or confrontational communication styles, exemplified by items like “My partner criticized me.” We adapted the instrument to measure changes in face-to-face communication patterns between the combatant and his partner. Participants were asked to compare their wartime communication (on both positive and negative scales) to their pre-war communication using a response scale ranging from 1 (decreased very much) to 5 (increased very much). Scoring for each subscale was calculated as the average of all item ratings within that subscale. A higher score on the Positive Communication scale suggests an improvement in communication during wartime compared to pre-war interactions. Conversely, a higher score on the Negative Communication scale indicates a deterioration in communication patterns during wartime. Cronbach’s alpha for internal consistency was α = 0.82 for the Positive subscale and α = 0.80 for the Negative subscale, demonstrating good reliability.

***The Background Questionnaire*** collected comprehensive demographic and personal information from participants, including age, employment status, relationship status, education level, parental status, religiosity, and other relevant details pertaining to their partner’s military service. For electronic communication, the participants were asked to indicate its frequency on a 7-point Likert scale, ranging from 1 (not at all) to 7 (several times a day), with intermediate points representing varying frequencies: 2 (a few times), 3 (once a month), 4 (once a week), 5 (several times a week), and 6 (once a day). For face-to-face communication, participants were asked to indicate its frequency when their combatant partner came home on leave, using a 5-point scale ranging from 1 (not at all since the beginning of the war) to 5 (every week or more), with intermediate points representing varying frequencies.

### Data analysis

The primary research hypotheses were tested using path analysis, a statistical method that examines relationships between variables to understand both direct and indirect effects, thereby providing a comprehensive view of the interconnected relationships within the model.

## Findings

### Descriptive analysis

We will first provide descriptive statistics of the main research variables, focusing on the dependent variable (mental health) by examining the distribution of its factors using an analysis of variance (ANOVA). We will then provide an overview of the independent variables (patterns of face-to-face and electronic communication), highlighting their distribution.

#### Mental health and its factors

A repeated measures ANOVA was conducted to examine differences in the levels of the three mental health factors: psychological, social, and emotional. Significant differences were found between the factors. The highest score was observed for psychological mental health, followed by social mental health, while the lowest score was for emotional mental health. Detailed means and standard deviations are presented in Table 1.

**Table 1.** Intercorrelations between main research variables.

Variables	1	2	3	4	5	6	7	8	*M*	*SD*
1. Emotional mental health	–								3.14	1.12
2. Social mental health	.57***	–							3.41	1.17
3. Psychological mental health	.62***	.48***	–						3.94	1.05
4. Social support	.39***	.34***	.48***	–					5.11	1.44
5. Positive electronic communication	.19**	.10	.16*	.36***	–				3.61	0.76
6. Negative electronic communication	–.12	.07	–.26***	–.24***	–.20**	–			2.70	0.82
7. Positive face to face communication shift	.11	.20***	.22**	.26***	.37***	–.28***	–		3.61	0.75
8. Negative face to face communication shift	–.24	–.13	–.17**	–.18**	–.25***	–.42***	–.25***	–	2.70	0.82

*p < .05, **p < .01, ***p < .001

#### Distribution of communication patterns between combatants and their partners

The distribution of communication patterns – both electronic and face-to-face – was examined to provide a comprehensive overview of the independent variables.

#### Electronic communication

To provide a comprehensive overview of the quality of electronic communication as measured by the ECI, we consolidated the categories from the original 5-point Likert scale into a new 3-level scale for both positive and negative aspects of communication: 1 (low frequency), 2 (medium frequency), and 3 (high frequency).

*Positive Communication*. 74.6% of the participants reported a high frequency of positive electronic communication, 14% reported a moderate frequency, and 11.4% reported a low frequency.

*Negative Communication*. 11.4% reported a high frequency of negative electronic communication, 20.5% reported a moderate frequency, and 68.1% reported a low frequency.

#### Face-to-face communication

To provide a comprehensive overview of the changes in the quality of face-to-face communication, we consolidated the categories from the original 5-point Likert scale into a new 3-level scale: greatly decreased, unchanged, and greatly increased.

##### Positive Communication

Among the participants, 50.6% reported an increase in positive communication compared to levels observed before the war. In contrast, 38.6% indicated that their communication remained unchanged, while 10.8% reported a decrease in positive communication.

##### Negative communication

Among the participants, 18.8% reported an increase in negative communication compared to levels observed before the war. In contrast, 47.8% indicated that their communication remained unchanged, while 33.4% reported a decrease in negative communication.

These findings reflect a complex evolution of communication dynamics during the war, highlighting notable increases in positive communication alongside an almost 20% increase in negative face-to-face communication compared to pre-war levels.

#### Correlations between research variables

Pearson correlation coefficients were calculated to explore the relationships between the main research variables. These analyses aimed to provide a detailed description of the main research variables and their interrelationships, serving as a foundation for the path analysis that will address the research questions. Social support was found to be positively correlated with all three components of mental health. Positive electronic communication showed a positive correlation with emotional mental health, while negative electronic communication was negatively correlated with psychological mental health. Positive face-to-face communication was positively correlated with social and psychological mental health, while negative face-to-face communication was negatively correlated with psychological mental health (see Table 1).

#### Path Model for explaining mental health factors

We developed two path analysis models to examine the research hypotheses and provide insights into mental health factors: the Electronic Communication Model, which examines interactions through electronic means, and the Face-to-Face Communication Model, which emphasizes changes in direct interpersonal communication compared to the pre-war period. The research variables were organized into four hierarchies, including exogenous variables, which are independent variables not explained by other variables in the model but may influence them, and endogenous variables, which are dependent variables explained by other variables within the model. Both models include four sets of variables introduced in a consistent order:

##### First hierarchy (exogenous variables - controls)

Exogenous variables included frequency of electronic communication, parental status (0 = no children, 1 = mothers), religious status (0 = secular, 1 = religious), and employment status (0 = not working, 1 = working). Other background variables (e.g., relationship status) were initially considered in preliminary analyses. However, those variables that were not significantly related to any of the endogenous variables were excluded from the final model. Only the variables significantly associated with the key outcomes were retained.

##### Second hierarchy (endogenous variables - communication channels)

In the Electronic Communication Model, these variables include positive and negative electronic communication. In the Face-to-Face Communication Model, they capture changes in positive and negative direct communication compared to pre-war levels.

##### Third hierarchy (endogenous variable - social support)

Social support was included as an endogenous variable to explore its mediating role in the relationships between different types of communication and mental health factors.

##### Fourth hierarchy (endogenous variables - outcome variables)

Outcome variables consist of the three components of mental health: emotional, social, and psychological well-being.

This structured approach allowed us to explore the associations between different types of communication, social support, and mental health.

## Empirical electronic communication model

**Figure 2.**
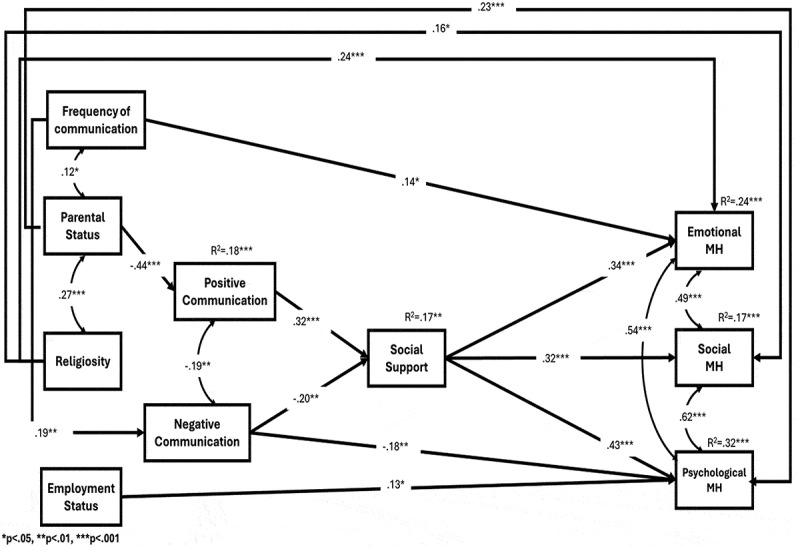
Empirical Model of electronic communication.

Significant direct and indirect relationships were observed between the independent variables and mental health factors (Tables 2 and 3, Figure 2).

**Table 2.** Path analysis for the electronic communication model - standardized coefficients of direct effects.

	Positive com.	Negative com.	Social support	Emotional mental health	Social mental health	Psychological mental health
Parental status	−.44*** (.07)	.06 (.08)		.03 (.08)	.16 (.09)	.23** (.07)
Religiosity	.12 (.07)	.06 (.08)		.24*** (.07)	.16* (.07)	.04 (.07)
Work status	.05 (.06)	−.05 (.08)		.05 (.06)	.07 (.06)	.13* (.07)
Communication frequency	.01 (.06)	.19** (.06)	.11 (.07)	.14* (.06)	.00 (.06)	.06 (.06)
Positive com.			.32*** (.07)	.08 (.08)	.05 (.09)	.07 (.06)
Negative com.	r=−.19* (.07)		−.20** (.07)	−.07 (.06)	−.01 (.07)	−.18** (.06)
Social support				.34*** (.07)	.32*** (.08)	.43*** (.06)
Emotional mental health						
Social mental health				r=.49*** (.06)		
Psychological mental health				r=.54*** (.06)	r=.62*** (.05)	
R^2^	.18** (.06)	.05 (.03)	.17** (.05)	.24*** (.05)	.17** (.05)	.32*** (.06)

*p < .05, **p < .01, ***p < .001.

CFI = 1.00, TLI = 1.00, Chi-Square = 3.46, df = 7, *p* = .840, RMSEA = .000, SRMR = .019.

comm. = communication.

**Table 3.** Path analysis of the electronic communication model - standardized indirect effect coefficients.

IV	M1	DV	IV → M	M → DV	IV → DV	Indirect	95% Indirect CI
Positive com.	Social support	Emotional mental health	.32*** (.07)	.34*** (.07)	.08 (.08)	.11*** (.03)	[.06, .18]
Negative com.	Social support	Emotional mental health	-.20** (.07)	.34*** (.07)	-.07 (.06)	-.07* (.03)	[-.14, -.02]
Positive com.	Social support	Social mental health	.32*** (.07)	.32*** (.08)	.05 (.09)	.10** (.04)	[.05, .19]
Negative com.	Social support	Social mental health	-.20** (.07)	.32*** (.08)	-.01 (.07)	-.06* (.03)	[-.13, -.02]
Positive com.	Social support	Psychological mental health	.32*** (.07)	.43*** (.06)	.07 (.06)	.14*** (.04)	[.07, .22]
Negative com.	Social support	Psychological mental health	-.20** (.07)	.43*** (.06)	-.18** (.06)	-.08* (.03)	[-.16, -.02]
Com. frequency	Negative com	Psychological mental health	.19** (.06)	-.18** (.06)	.06 (.06)	-.03* (.02)	[-.08, -.01]

IV	M1	M2	DV	IV → M1	M1 → M2	M2 → DV	IV → DV	Indirect	95% Indirect CI
Com. frequency	Negative com.	Social support	Emotional mental health	.19** (.06)	-.20** (.07)	.34*** (.07)	.14* (.06)	-.01* (.007)	[-.03, -.003]
Com. frequency	Negative com.	Social support	Social mental health	.19** (.06)	-.20** (.07)	.32*** (.08)	.00 (.06)	-.01* (.006)	[-.03, -.003]
Com. frequency	Negative com.	Social support	Psychological mental health	.19** (.06)	-.20** (.07)	.43*** (.06)	.06 (.06)	-.02* (.008)	[-.04, -.004]

*p < .05, **p < .01, ***p < .001.

com. = communication, DV = Dependent variable, *M* = Mediator, IV = Independent variable.

### Paths between background variables and endogenous variables

The background variables included parental status, religious status, employment status, and frequency of electronic communication. The paths between each background variable and the endogenous variables are described below.

#### Parental status

Significant differences in communication and mental health factors were found based on parental status. Women with children reported lower levels of positive electronic communication compared to those without children. However, they reported higher levels of psychological mental health.

#### Religious status

Religious status was positively associated with emotional and social mental health. Religious participants reported higher levels of these mental health factors than their secular counterparts. However, no significant relationship was observed between religious status and psychological mental health.

#### Employment status

Employment status was linked to psychological mental health. Working participants exhibited higher levels of psychological well-being compared to those who were not working.

#### Frequency of electronic communication

Yielded mixed associations with communication and mental health. Higher frequency was associated with increased negative communication with partners. However, frequent electronic communication was linked to better emotional mental health. No significant relationships were found between the frequency of electronic communication and social or psychological mental health.

### Paths between endogenous variables

#### Direct effects (examining hypotheses 1–3 for the electronic communication model)

The analysis identified significant direct effects between variables. A positive relationship was observed between positive electronic communication and social support, with higher ratings of positive communication associated with greater levels of social support. Conversely, negative electronic communication showed a negative relationship with social support, where higher levels of negative communication corresponded to lower levels of social support (Table 2). Social support itself was positively related to all three mental health factors emotionally, social, and psychological. These findings support Hypothesis 1 for the Electronic Communication Model.

Importantly, no direct paths were identified between positive communication and the three mental health factors, meaning that Hypothesis 2 was not supported. No negative relationship was found between negative communication and mental health factors. Thus, Hypothesis 3 was not confirmed for the Electronic Communication Model.

**Indirect effects *(examining hypotheses 4–5 for the electronic communication model)***

The analysis revealed several significant indirect paths between communication patterns and mental health factors, mediated by social support (Table 3, Figure 2).

*Mediation: Positive communication, social support, and mental health*. Social support mediated the relationship between positive electronic communication and all three mental health factors. Positive electronic communication was related to higher social support, which in turn was related to higher scores for all three mental health factors. This finding supports Hypothesis 4 for the Electronic Communication Model.

*Mediation: Negative communication, social support, and mental health*. A similar pattern was observed for negative electronic communication. Social support mediated the relationship between negative communication and the three factors of mental well-being. Higher levels of negative communication were related to lower social support, which in turn was related to lower scores for all three mental health factors. This result confirms Hypothesis 5 for the Electronic Communication Model.

In conclusion, the highest proportion of explained variance was observed for psychological mental health, followed by emotional and social mental health.

## Empirical Face-to-face communication model

### Paths between background and endogenous variables

The relationships between background variables and endogenous variables in the Face-to-Face Communication Model were found to align with those observed in the Electronic Communication Model.

### Direct effects (examining hypotheses 1–3 for the face-to-face communication model)

**Figure 3.**
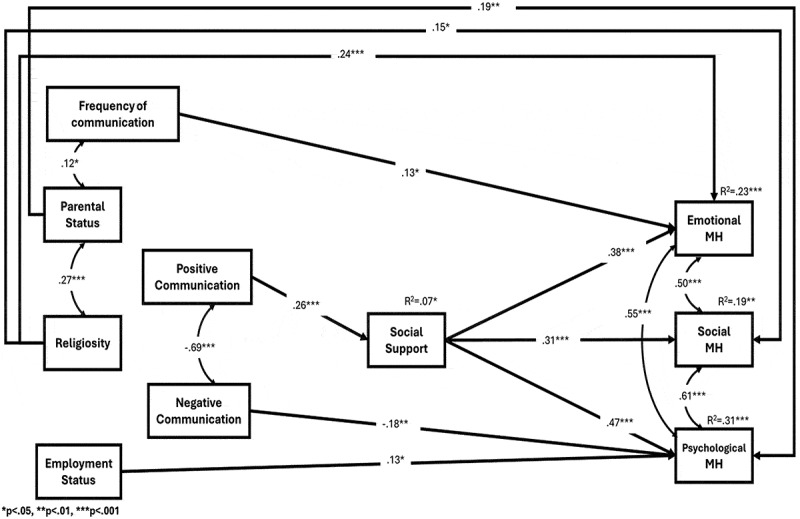
Empirical model of face-to face communication.

The analysis yielded a significant positive relationship between increases in positive face-to-face communication compared to the pre-war situation, and social support. Greater increases in positive communication were associated with higher levels of social support (Table 4, Figure 3). Social support, in turn, was positively related to emotional, social, and psychological mental health, confirming Hypothesis 1 for the Face-to-Face Communication Model.

**Table 4.** Path analysis of the face-to-face communication model - standardized direct effect coefficients .

	Positive com.	Negative com.	Social support	Emotional mental health	Social mental health	Psychological mental health
Parental status	−.06 (.08)	−.08 (.08)		−.002 (.07)	.14 (.08)	.19** (.06)
Religiosity	.11 (.07)	.04 (.07)		.24*** (.07)	.15* (.07)	.03 (.07)
Work status	.13 (.07)	−.12 (.08)		.06 (.06)	.06 (.06)	.13* (.06)
Com. frequency	.03 (.07)	.07 (.07)	.05 (.07)	.13* (.06)	−.01 (.06)	.02 (.06)
Positive com.			.26** (.10)	−.01 (.10)	.12 (.11)	.07 (.09)
Negative com.	r = −.69*** (.04)		−.01** (.10)	.01 (.10)	.02 (.11)	−.01 (.10)
Social support				.38*** (.06)	.31*** (.07)	.47*** (.06)
Emotional mental health						
Social mental health				r = .50*** (.06)		
Psychological mental health				r = .55*** (.06)	r = .61*** (.05)	
R^2^	.03 (.03)	.02 (.03)	.07* (.04)	.23*** (.05)	.19** (.06)	.31*** (.06)

*p < .05, **p < .01, ***p < .001.

CFI = 1.00, TLI = 1.00, Chi-Square = 3.52, df = 7, *p* = .83, RMSEA = .000, SRMR = .016.

com. = communication.

No direct paths were identified between positive communication and the mental health factors, failing to confirm Hypothesis 2 for the Face-to-Face Communication Model. However, a negative relationship was observed between negative communication and psychological mental health, indicating that increases in negative communication are associated with lower psychological mental health. This finding partially supports Hypothesis 3 for the Face-to-Face Communication Model.

### Indirect effects (examining hypotheses 4–5 for the face to face communication model)

The analysis revealed several significant indirect paths in the Face-to-Face Communication Model (Table 5, Figure 3).

**Table 5.** Path analysis of the face-to-face communication model - standardized indirect effect coefficients.

IV	M	DV	IV → M	M → DV	IV → DV	Indirect	95% Indirect CI
Positive com.	Social support	Emotional mental health	.26** (.10)	.38*** (.06)	−.01 (.10)	.10* (.04)	[.02, .20]
Positive com.	Social support	Social mental health	.26** (.10)	.31*** (.07)	.12 (.11)	.08* (.04)	[.02, .17]
Positive com.	Social support	Psychological mental health	.26** (.10)	.47*** (.06)	.07 (.09)	.12** (.05)	[.03, .23]

*p < .05, **p < .01, ***p < .001.

com. = communication, DV = Dependent variable, *M*= Mediator, IV = Independent variable.

*Mediation: Positive communication, social support, and mental health*. Social support mediated the relationship between positive face-to-face communication and all three mental health factors. Increases in positive communication were related to higher social support, which in turn was related to higher levels of emotional, social, and psychological mental health. These findings support Hypothesis 4 for the Face-to-Face Communication Model.

*Mediation: Negative communication, social support, and psychological mental health*. Partial mediation was observed for negative communication. Social support mediated the relationship between negative face-to-face communication and psychological mental health. Increases in negative communication were related to lower levels of social support, which in turn was related to lower psychological mental health. This result partially confirms Hypothesis 5 for the Face-to-Face Communication Model.

In conclusion, the highest proportion of explained variance was observed for psychological mental health, followed by emotional and social mental health.

### Comparison of relationship paths between the two models

Comparison of path coefficients between the two communication models revealed both similarities and differences in the patterns of relationships between communication types, social support, and mental health factors.

#### Similarities

In both models, background variables showed similar path coefficients in explaining mental health. Social support exhibited positive path coefficients with all three mental health factors in both contexts. Positive communication between partners, through electronic channels or as an increase in face-to-face communication compared to the pre-war situation, showed indirect positive path coefficients with mental health through social support. Negative communication, in both electronic and face-to-face contexts, displayed direct negative path coefficients with psychological mental health

#### Differences

A key distinction between the two models is that negative electronic communication yielded a negative path coefficient with social support, a pattern not observed with increased negative face-to-face communication. Negative electronic communication exhibited both direct and indirect path coefficients with mental health through social support, while increased negative face-to-face communication only showed a direct path coefficient. These findings underscore the nuanced impact of different communication patterns on mental health factors.

## Discussion

Several key findings of this study deserve to be mentioned, as they shed light on the intricate connections between communication patterns, social support, and mental health among combatants’ partners. First, background factors like parental status, religious affiliation, and employment status showed associations with various mental health outcomes. Furthermore, stronger social support was consistently linked to greater emotional, social, and psychological well-being. Positive communication, whether through electronic channels or in person, indirectly contributed to better mental health by bolstering social support networks. Conversely, negative communication was directly associated with poorer psychological well-being. Perhaps most interestingly, the analysis revealed a negative association between negative electronic communication and social support, a relationship unique to electronic communication and not observed in face-to-face communication. Taken together, these findings underscore the complex and nuanced ways that different communication patterns can impact the mental health of partners of combatants during times of conflict, suggesting avenues for targeted interventions and support.

Mental health emerged as a multifaceted construct, with partners experiencing varying levels across its different dimensions simultaneously. For example, emotional mental health tended to be relatively low due to the stress and uncertainty of war, whereas psychological resilience was notably higher. The differences in levels of mental health factors can be attributed to the complex nature of human responses to crises. While the stress and uncertainty of war naturally lead to decreased emotional mental health, challenging circumstances can also activate coping mechanisms and foster resilience, reflected in relatively higher levels of psychological mental health (Tedeschi & Calhoun, 2004). This phenomenon, known as stress-related growth or adversity-activated development, suggests that individuals can develop increased strength, self-reliance, and new perspectives on life in the face of adversity (Joseph & Linley, 2006). In this vein, Bonanno’s (2004) research on resilience in the face of loss and trauma further revealed that individuals often maintain relatively stable psychological and physical functioning when confronted with potentially stressful events.

In terms of communication quality, interactions between combatants and their partners were generally positive across channels. Nonetheless, approximately 20% of the participants reported an increase in negative face-to-face communication compared to the pre-war period. This may raise concerns about the state of some couple relationships in the aftermath of war. These findings suggest the need for tailored support that addresses specific mental health dimensions, an understanding of factors contributing to resilience, and the importance of relationship support services to navigate the challenges posed by wartime experiences. As we delve deeper into these findings, it is essential to discuss the specific research hypotheses that guided this study, providing insight into partners’ relationships during wartime.

### Impacts of background and key research variables on mental health

The complex relationships between background variables, key research variables (communication channels and social support), and mental health factors during wartime reveal intriguing patterns among combatants’ partners. Background variables, such as parental status, religiosity, and employment status, showed consistent effects across both types of communication models. Having children was positively correlated with better psychological health, consistent with Kronfeld’s (2000) finding that mothers with multiple children adapted better to personal crises, such as breast cancer, compared to mothers with fewer children or those without children. According to Kronfeld, this adaptation may stem from an enhanced sense of purpose and responsibility, driven by the need to care for and support their children, as well as the potential for a stronger support network provided by a larger family. Higher religiosity was linked to improved emotional and social mental health, aligning with a broader body of research indicating that higher levels of religiosity are often associated with better mental health outcomes and subjective well-being (Abdel-Khalek et al., 2019; Zimmer et al., 2016). Furthermore, the communal nature of Judaism seems to foster a sense of security and calmness, enhancing social health (Levin, 2013). Paid work also enhances psychological health, with Kulik and Ramon (in press) noting that maintaining routines benefits working mothers coping with stress, likely by providing a sense of stability and normality.

Regarding the process variable, the frequency of electronic communication showed complex effects. Despite findings that frequent electronic communication can negatively impact perceived communication quality, it was positively related to partners’ emotional health. This paradox suggests that while frequent electronic communication might lead to sharing more negative content, the act of staying connected offers emotional comfort. This is in line with research on long-distance relationships and military families, showing that regular communication can help reduce uncertainty and provide a sense of closeness crucial for maintaining emotional well-being during separations (Carter & Renshaw, 2016; Maguire & Kinney, 2010).

### Relationships between social support, communication, and mental health

The findings show that social support is significantly related to the three factors of mental health among combatants’ partners in both models of communication (confirming Hypothesis 1). This aligns with research findings by Padden et al. (2011), which demonstrate that social support during times of war is connected to mental health outcomes among combatants’ partners. Negative communication between combatants and their partners was negatively related to the partners’ psychological mental health, but not to their social and emotional mental health in both communication channels (partially confirming Hypothesis 3). Arguments, unpleasant interactions, disagreements, or criticism from combatants lowered their partners’ sense of self-worth, directly affecting their psychological resilience and strength during wartime. This differential impact on various aspects of mental health reveals a nuanced interplay between communication patterns and psychological well-being.

The specific effect on psychological mental health, as opposed to social and emotional dimensions, may be attributed to the cognitive nature of psychological resilience and strength. Negative communication likely challenges one’s self-perception and coping mechanisms, which are fundamental components of psychological health. This suggests that the cognitive processes involved in maintaining psychological resilience in high-stress situations may be particularly susceptible to negative verbal inputs, especially from significant others. The absence of a significant relationship between communication patterns and social or emotional mental health is noteworthy. It might indicate that the emotional and social aspects of mental health are more resilient to short-term negative interactions, possibly due to established coping mechanisms or external support systems.

Positive communication in both channels was not directly related to mental health (rejecting Hypothesis 2). This connection existed only through the mediation of social support (confirming Hypothesis 4). It may be concluded that contrary to common belief, positive communication did not necessarily benefit combatants’ partners in the unique context of war due to the diverse stressors they encounter during the combatants’ deployment (Everson et al., 2017). Moreover, positive electronic communication, which occurs at a distance and does not allow for physical intimacy, could intensify feelings of absence and longing (Lapp et al., 2010). As for positive communication, several dynamics in the partner-combatant interaction can diminish its potential beneficial impact on partners’ mental health during military deployment. During leave periods, service members’ thoughts are often focused on the battlefield and their comrades. This preoccupation may limit their emotional availability to their partners, thereby reducing the positive impact of communication on the partners’ mental health, especially when partners expect attention and support from their deployed loved ones (Knobloch et al., 2013).

Differences were found between the two models in the pathways between negative communication and mental health. In electronic communication, negative communication was negatively related to social support, indicating that disagreement and criticism affected participants’ assessment of social support. This relationship was not found in face-to-face communication (partially confirming Hypothesis 5). The holistic nature of face-to-face interactions, allowing for touch and expressions of intimacy, may compensate for negative evaluations, preserving the perception of social support even in cases of negative communication between the partners.

In conclusion, face-to-face communication between combatants and their partners is more complex than electronic communication, involving negative, positive, and compensatory effects that contribute to diverse experiences among combatants’ partners. These findings highlight the nuanced interplay between communication channels, social support, and mental health in the context of relationships with combatants during wartime.

## Conclusions and contribution

The study presents several important conclusions and contribution regarding communication between partners during wartime, revealing that communication (both electronic and face-to-face) was generally positive. However, approximately 20% of participants reported an increase in negative face-to-face communication compared to pre-war situations, suggesting potential harmful consequences for relationships in the postwar period. Negative communication was found to have a direct harmful impact on partners’ mental health, whereas positive communication only affected mental health when linked to social support. This finding suggests that negative communication has a more apparent detrimental effect than positive communication.

Moreover, it was found that resources available during crises, such as wartime, do not operate independently but create a continuum of effects that benefit individuals in a chain reaction. This is evident in the lack of a direct relationship between positive communication and mental health, except when mediated by social support. These findings highlight a significant advancement in COR theory through Hobfoll’s (2014) concept of resource caravans, emphasizing the interconnected nature of resources. Specifically, positive communication is positively associated with social support, which is directly linked to various mental health factors. Thus, social support serves as a crucial resource, mediating the relationship between positive communication and mental health. This mediating role suggests that social support creates a resource caravan passageway, amplifying the effects of positive communication on mental health.

### Research limitations and recommendations for future research

One limitation of this study lies in the measurement of changes in face-to-face communication, which relied on participants’ recollections and comparisons of communication quality before and during the war. This reliance on memory may have introduced recall bias, as participants’ perceptions of pre-war communication could be influenced by their current experiences and emotions during the war. Another limitation is the cross-sectional design, where data were collected at a single point in time. This makes it difficult to determine the direction of the relationship between variables. It is possible that mental health influences interpersonal communication rather than vice versa. Future studies should employ longitudinal designs to better establish causal relationships and examine the impact of communication on mental health at various stages of war. Researchers should first assess the quality of communication between partners and then, several months later, evaluate their mental health. It is important to note that this study was conducted between the fourth and fifth months of the war, during a stage of “war routine.” The findings and conclusions are thus specific to this stage. Future research should examine mental health and communication patterns at different war stages, including initial deployment, mid-deployment, and post-deployment.

Another limitation of this study is the sampling method, which involved selecting participants from a support group for combatants’ partners. This approach may introduce bias, as women involved in such groups might possess unique characteristics that distinguish them from other partners of combatants. Their experiences may not fully represent the broader population of combatants’ partners. Women who do not participate in support groups may face different challenges and potentially experience higher levels of psychological distress due to the lack of focused support from peers in similar situations. The absence of access to dedicated social support could negatively affect their mental health, as social support is a well-established factor in promoting mental well-being. Future research should aim to include a more diverse sample of women whose partners are combatants to provide a more comprehensive understanding of their experiences and the impact on their mental health. In this vein, given that cultural norms may influence communication patterns between partners, future research examining the relationship between communication between combatants and their partners in multicultural societies should shed light on cultural differences that shape these communication patterns.

This study focused on examining communication between male combatants and their female partners. However, since in many countries, women also serve as combatants (e.g., Israel, the United States, Canada, Norway), future research should explore the impact of communication patterns in different types of couples according to the combatant’s gender. Specifically, studies should investigate communication among female combatants and their male partners who are not combatants, as well as cases where both partners are combatants. Examining communication patterns in different types of couples can provide valuable insights into how communication affects mental well-being and relationship quality. Finally, to gain a comprehensive understanding of partners’ communication patterns during wartime and their impact on well-being, future research should combine quantitative and qualitative methods. This mixed-methods approach could include in-depth interviews or focus groups to complement quantitative data and provide richer insights into the experiences of women whose partners are combatants. Investigating the role of military culture in shaping communication patterns and help-seeking behaviors could provide valuable insights for developing targeted interventions. Understanding how military culture influences these aspects can help tailor more effective and culturally sensitive support systems.

### Practical recommendations

Couples experiencing wartime separation should be mindful that frequent electronic communication might lead to discussions on unresolved issues that arise due to the challenges of war, increasing distress and potentially harming communication patterns. To address this, partners should be aware of the dynamics that develop as communication frequency increases and avoid unnecessary criticism or demands. It is crucial for both partners to recognize the potential negative impact of frequent communication and cultivate support networks outside their relationship to reduce over-reliance on each other for emotional support. Couples should develop emotional awareness, particularly regarding the potential spillover of emotions arising during wartime – whether from the battlefield for combatants or from daily coping challenges faced by their partners – into their communication and interactions. Emphasizing self-care and maintaining a sense of purpose outside the relationship can also contribute to resilience.

Therapists working with military couples should emphasize fostering open and constructive communication, creating a safe space for partners to share experiences and collaboratively seek solutions. Encouraging dialogue that avoids criticism or blame can help couples address difficult topics more effectively. Developing effective interpersonal skills is key to managing the emotional challenges of military service while preserving the strength of their relationship. By building a resilient support system – comprising both the couple’s relationship dynamics and external professional resources such as counseling, peer support groups, and community services – couples can better navigate the unique pressures of wartime, safeguard their mental health, and enhance their overall well-being.

## Data Availability

The data that support the findings of this study are not publicly available due to privacy and ethical restrictions. The data contain information that could compromise the privacy of research participants. Researchers who meet the criteria for access to confidential data may request access from the corresponding author, subject to approval from our institutional review board and under strict confidentiality agreements.

## Data deposition Statement

Due to the sensitive nature of the research data, which includes personal information of study participants, the data underlying this article have not been deposited in a public repository. Qualified researchers may request access to de-identified data by contacting the corresponding author. Any data sharing will be subject to institutional review board approval and will require a formal data sharing agreement to ensure participant confidentiality.
